# Distributed Neural Plasticity for Shape Learning in the Human Visual Cortex

**DOI:** 10.1371/journal.pbio.0030204

**Published:** 2005-06-07

**Authors:** Zoe Kourtzi, Lisa R Betts, Pegah Sarkheil, Andrew E Welchman

**Affiliations:** **1**Max-Planck Institute for Biological CyberneticsTübingenGermany; **2**School of Psychology, University of BirminghamUnited Kingdom; **3**McMaster UniversityOntarioCanada; National Institute of Mental HealthUnited States of America

## Abstract

Expertise in recognizing objects in cluttered scenes is a critical skill for our interactions in complex environments and is thought to develop with learning. However, the neural implementation of object learning across stages of visual analysis in the human brain remains largely unknown. Using combined psychophysics and functional magnetic resonance imaging (fMRI), we show a link between shape-specific learning in cluttered scenes and distributed neuronal plasticity in the human visual cortex. We report stronger fMRI responses for trained than untrained shapes across early and higher visual areas when observers learned to detect low-salience shapes in noisy backgrounds. However, training with high-salience pop-out targets resulted in lower fMRI responses for trained than untrained shapes in higher occipitotemporal areas. These findings suggest that learning of camouflaged shapes is mediated by increasing neural sensitivity across visual areas to bolster target segmentation and feature integration. In contrast, learning of prominent pop-out shapes is mediated by associations at higher occipitotemporal areas that support sparser coding of the critical features for target recognition. We propose that the human brain learns novel objects in complex scenes by reorganizing shape processing across visual areas, while taking advantage of natural image correlations that determine the distinctiveness of target shapes.

## Abstract 2

Learning to recognize objects involves distinct neural changes in several visual cortical areas.

## Introduction

Expertise in detecting and recognizing objects in natural scenes, where targets are camouflaged by their backgrounds, is critical for many of our interactions in complex environments: from identifying predators or prey and recognizing poisonous foods, to diagnosing tumors on medical images and finding familiar faces in the crowd. As with many skills, learning has been shown to be a key facilitator in the detection and recognition of targets in cluttered scenes [1–8]. Previous neurophysiological [9–15] and imaging [16–19] studies on object learning have concentrated on the higher stages of visual (inferior temporal cortex) and cognitive processing (prefrontal cortex), providing evidence that the representations of shape features in these areas are modulated by learning. In contrast, computational approaches have proposed that associations between features that mediate the recognition of familiar objects may occur across different stages of visual analysis, from orientation detectors in the primary visual cortex to occipitotemporal neurons tuned to object parts and views [20–22]. However, the neural implementation of object learning mechanisms across stages of visual analysis is largely unknown, and the question of how the visual brain learns objects in natural cluttered scenes remains open.

**Figure 1:**
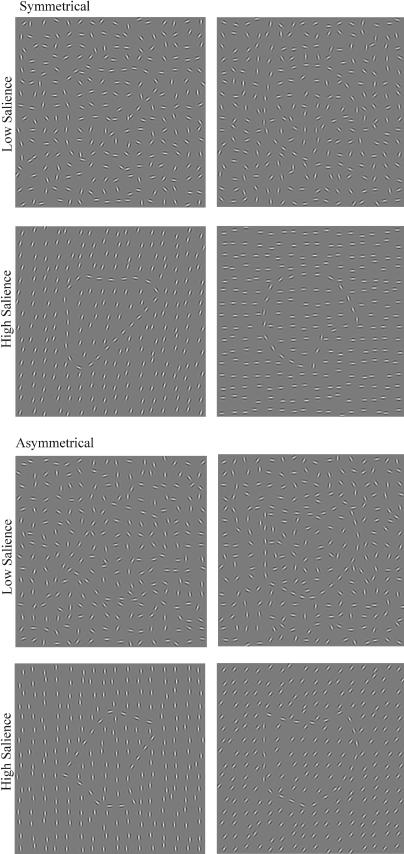
Stimuli Examples of symmetrical and asymmetrical low- and high-salience stimuli.

The aim of our study was 2-fold: (1) to investigate the neural plasticity mechanisms that mediate shape learning in cluttered scenes across stages of visual processing in the human visual cortex, and (2) to examine the effect of regularities present in natural scenes (i.e., grouping of similar features) that determine the distinctiveness of targets in noisy backgrounds (i.e., perceptual saliency) on this learning-dependent plasticity. To this end, we used human functional magnetic resonance imaging (fMRI) combined with psychophysics. To gain insight into the neural mechanisms that mediate shape-specific learning, we examined fMRI responses evoked when observers detected shapes that they had learned through training compared with responses evoked when observers detected shapes on which they had not been trained. To investigate the effects of learning in the detection of visual shapes in cluttered scenes, we manipulated the salience of the target shapes by altering their distinctiveness from the background ( Figure 1). We compared behavioral performance and fMRI responses for low-salience shapes in noise (Experiment 1) and high-salience pop-out targets (Experiment 2).

Our stimuli consisted of shapes defined by a closed contour of similarly oriented Gabor elements that were embedded in a background of Gabor elements. These stimuli (see Figure 1) yield the perception of a global figure in a textured background rather than simple paths (i.e., open contours). These aligned contours have been shown to result from the integration of the similarly oriented elements into global configurations [23–25]. Previous work has shown that these stimuli involve processing in both early retinotopic and higher occipitotemporal regions [26]. In Experiment 1, observers were presented with low-salience stimuli in which shapes were embedded in a background of randomly positioned and oriented Gabors. In Experiment 2, high-salience stimuli were used in which shapes were embedded in a background of randomly positioned, but uniformly oriented Gabors. In both experiments, observers were required to decide which of two shapes presented on either side of the central fixation point was symmetrical. Initially, observers performed this task in the scanner with two sets of untrained stimuli. Observers were then trained in the laboratory with feedback on three consecutive days on one set of stimuli, and then tested again in the scanner with the trained set and the originally presented, untrained set of stimuli ( Figure 1).

**Figure 2:**
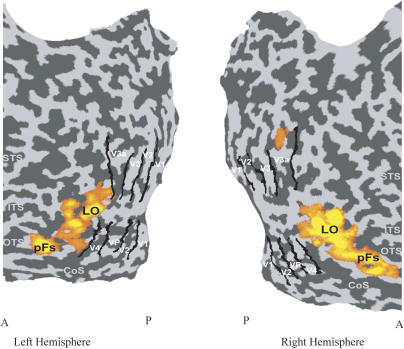
ROIs Functional activation maps for one subject showing the early retinotopic ventral (V1, V2, VP, and V4) and dorsal (V1, V2, V3, and V3a) areas and the LOC subregions. Functional activations (color-maps) are superimposed on flattened cortical surfaces of the right and left hemispheres. A, anterior; CoS, collateral sulcus; ITS, inferior temporal sulcus; OTS, occipitotemporal sulcus; P, posterior; STS, superior temporal sulcus.

Our findings suggest a link between shape-specific perceptual learning and neural plasticity mechanisms in the human visual cortex. Specifically, for low-salience shapes, improved behavioral performance was coupled with increased fMRI responses for trained shapes in early retinotopic areas (V1, V2, Vp, and V4v) and the lateral occipital complex (LOC), a region in the lateral occipital cortex extending anterior in the temporal cortex ( Figure 2) that is thought to be involved in the analysis of object shape [27] and processes of object recognition [19, 27]. In contrast to the increased responses for trained low-salience shapes, when observers learned high-salience shapes, lower fMRI responses were evoked in the LOC, and no evidence for plasticity in early retinotopic visual areas was observed. These findings provide novel evidence for distributed neural plasticity mechanisms across stages of visual analysis that are adaptable to image regularities which determine the perceptual saliency of targets in cluttered scenes ( Figure 2).

## Results

### Experiment 1: Learning Low-Salience Shapes

#### Behavioral performance

**Figure 3:**
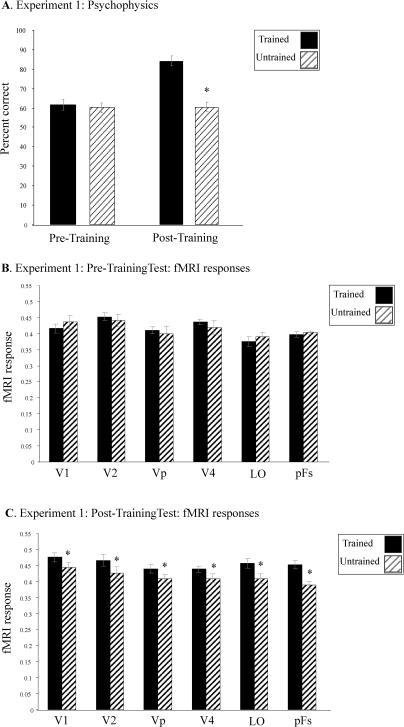
Results for Experiment 1 Psychophysical data (A) and fMRI responses obtained during the scanning sessions before (B) and after (C) training. Error bars indicate the SEM across subjects. Significant differences are indicated by asterisks. (A) Psychophysical data (percent correct) are shown for trained and untrained shapes in the tests before and after training. Normalized fMRI responses across subjects for trained and untrained shapes before (B) and after (C) training across the LOC subregions and the early ventral areas. Normalized fMRI responses were computed by subtracting the mean signal (percent signal change from fixation baseline) across conditions, sessions, and ROIs from the signal in each condition per subject and adding the overall average across conditions, sessions, ROIs, and subjects. These normalized fMRI responses indicate differences across conditions independent of the variability in the fMRI signal across subjects, scanning sessions, and ROIs.

In experiment 1 we examined behavioral performance (accuracy in detecting symmetrical shapes) and fMRI responses when observers were trained with low-salience shapes. Figure 3A shows the behavioral performance of the observers for trained and untrained stimuli during the scanning sessions before and after training. Before training, observers' performance was similar for the set of stimuli that would become familiar through training and for the set of stimuli that would remain untrained. This was expected because both sets of stimuli were novel before training. However, after training, there was a significant increase in performance for the trained, but not the untrained, stimuli. Specifically, a repeated measures analysis of variance (ANOVA) showed the main effects of test session (before and after training) (F_1,10_ = 15.81, *p* < 0.01) and familiarity (trained and untrained) (F_1,10_ = 81.63, *p* < 0.001) and a significant interaction between these variables (F_1,10_ = 66.76, *p* < 0.001).

#### Pre-training and post-training fMRI data

For each individual subject, we identified the early visual areas, posterior (lateral occipital [LO]) and anterior (posterior fusiform sulcus [pFs]) subregions of the LOC (see Figure 2), as cortical regions of interest (ROIs) in which we examined fMRI responses before and after training. Before training, fMRI responses in these regions were not different between the two sets of shapes (those to become trained vs. those to remain untrained) ( Figure 3B). Specifically, a repeated measures ANOVA showed no effect of familiarity (F_1,50_ < 1, *p* = 0.65). Again, this is not surprising because the subjects have not been trained on any shapes. However, after subjects had been trained on the low-salience shapes, significantly stronger fMRI responses were observed for trained than untrained shapes ( Figure 3C). This was true in both the early visual areas (F_1,50_ = 7.02, *p* < 0.05) and the LOC subregions (LO: (F_1,50_ = 10.92, *p* < 0.001), pFs: (F_1,50_ = 5.85, *p* < 0.01)). Further, comparison of the fMRI responses before and after training showed a significant (F_1,50_ = 3.06, *p* < 0.05) interaction between test session (before and after training) and familiarity (trained and untrained). That is, we observed significantly stronger responses for the trained shapes (F_1,50_ = 3.58, *p* < 0.05) after than before training, but no significant differences for the untrained shapes (F_1,50_ < 1, *p* = 0.41) ( Figure 3).

### Experiment 2: Learning High-Salience Shapes

#### Behavioral performance

**Figure 4:**
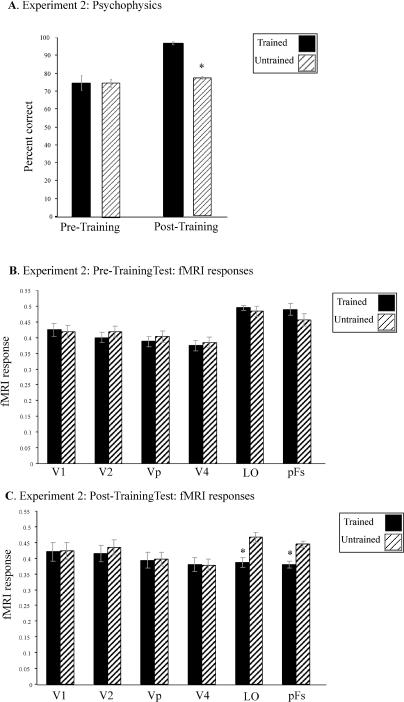
Results for Experiment 2 Psychophysical data (A) and fMRI responses obtained during the scanning sessions before (B) and after (C) training. Error bars indicate the SEM. Significant differences are indicated by asterisks. (A) Psychophysical data (percent correct) for trained and untrained shapes in the tests before and after training. Normalized fMRI responses across subjects for trained and untrained shapes before (B) and after (C) training across the LOC subregions and early ventral areas.

In Experiment 2, we examined behavioral performance and fMRI responses when observers were trained with high-salience shapes. Figure 4A shows the behavioral performance of the observers for trained and untrained stimuli during the scanning sessions before and after training. Before training on high-salience shapes, there was no difference in performance when testing with the different stimulus sets (those to become trained vs. those to remain untrained). However after training, observers showed a significant improvement for the trained shapes compared with the untrained shapes. Specifically, a repeated measures ANOVA showed main effects of test session (F_1,7_ = 80.98, *p* < 0.001), familiarity (F_1,7_ = 37.70, *p* < 0.001), and a significant interaction for test session and familiarity (F_1,7_ = 39.05, *p* < 0.001).

#### Pre-training and post-training fMRI data

As shown in Figure 4B, and as expected, no differences were observed before training between the fMRI responses to shapes that would become trained and those that would remain untrained. That is, there was no significant effect for familiarity (F_1,35_ < 1, *p* = 0.82) before training. In contrast to the results from Experiment 1, after training we found lower fMRI responses for trained compared to untrained stimuli for high-salience shapes ( Figure 4C). A further difference from Experiment 1 was that this learning effect was evident in the LOC, but not in early visual areas. Specifically, a repeated measures ANOVA showed significantly stronger responses for untrained than trained shapes in the LOC subregions (LO: (F_1,35_ = 13.73, *p* < 0.01), pFs: (F_1,35_ = 5.21, *p* < 0.05)) but not in early visual areas (F_1,35_ < 1, *p* = 0.38). Further, comparison of the fMRI responses before and after training showed a significant interaction between test session and ROI (F_1,35_ = 3.87, *p* < 0.01), and familiarity and ROI (F_1,35_ = 3.11, *p* < 0.05). That is, in the LOC we observed significantly lower responses for the trained shapes (F_1,35_ = 27.43, *p* < 0.001) after than before training, but no significant differences for the untrained shapes (F_1,35_ = 1.58, *p* = 0.19). In the early visual areas, no significant differences were observed for trained (F_1,35_ = 2.92, *p* = 0.13) or untrained (F_1,35_ = 2.77, *p* = 0.14) shapes before and after training ( Figure 4).

### Comparison across Experiments

**Figure 5:**
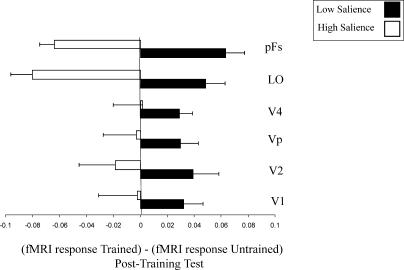
Summary of Results fMRI learning effects for low-salience (Experiment 1) and high-salience (Experiment 2) shapes indicated by subtracting the fMRI responses for untrained from those for trained stimuli in the post-training session in each experiment. Error bars are plus or minus the SEM. Positive values indicate stronger fMRI responses for trained stimuli, whereas negative values indicate stronger fMRI responses for untrained stimuli.

In Figure 5, we summarize the fMRI learning effects after training for low-salience (Experiment 1) and high-salience (Experiment 2) stimuli, by plotting the differences between fMRI responses for trained and untrained stimuli in the post-training session across visual areas. This analysis showed positive differences (stronger fMRI responses for trained than untrained stimuli) for low-salience shapes across visual areas, whereas negative differences (stronger fMRI responses for untrained than trained stimuli) for high-salience shapes in the LOC.

**Figure 6:**
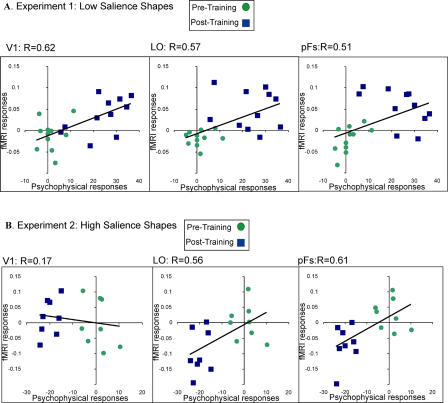
Relationship between Psychophysical and fMRI Learning Effects fMRI data and the corresponding psychophysical response for low-salience (A) and high-salience (B) shapes. For each individual subject, we plotted a behavioral learning index (percent correct for trained minus percent correct for untrained stimuli) and an fMRI learning index (percent signal change for trained minus percent signal change for untrained stimuli) after training. Positive values indicate stronger responses for trained than untrained shapes, whereas negative values indicate lower responses for trained than untrained shapes. For low-salience shapes the regression analysis was significant in early visual areas (V1: *r* = 0.62, F_1,21_ = 13.65, *p* = 0.001; V2: *r* = 0.42, F_1,21_ = 6.65, *p* < 0.05; Vp: *r* = 0.50, F_1,21_ = 7.62, *p* = 0.01; V4: *r* = 0.50, F_1,21_ = 8.91, *p* < 0.01) and the LOC subregions (LO: *r* = 0.57, F_1,21_ = 9.84, *p* < 0.01; pFs: *r* = 0.51, F_1,21_ = 7.18, *p* = 0.01). For high-salience shapes the regression was significant only in the LOC subregions (LO: *r* = 0.56, F_1,15_ = 6.69, *p* < 0.05; pFs: *r* = 0.61, F_1,15_ = 8.58, *p* = 0.01) but not in the early visual areas (V1: *r* = 0.17, F_1,15_ < 1, *p* = 0.51; V2: *r* = 0.25, F_1,15_ < 1, *p* = 0.34; Vp: *r* = 0.28, F_1,15_ = 1.27, *p* = 0.27; V4: *r* = 0.15, F_1,15_ < 1, *p* = 0.56).

To further quantify the relationship between the behavioral and fMRI learning effects, we conducted a regression analysis on the psychophysical and fMRI responses from individual subjects across visual areas for Experiments 1 and 2. This analysis provides additional evidence for a link between behavioral improvement and neuronal changes after training; that is, higher differences between trained and untrained stimuli were observed in both the behavioral and fMRI responses after than before training. As shown in Figure 6, for low-salience shapes, this regression analysis was significant in early visual areas (V1 is shown as a representative area, but see figure caption for other areas) and the LOC subregions, whereas for high-salience shapes, the regression was significant only in the LOC subregions. The majority of positive points in the plots for low-salience shapes indicates stronger responses for trained than untrained shapes after training, whereas the majority of negative points for high-salience shapes indicates lower responses for trained than untrained shapes after training ( Figure 6).

**Figure 7:**
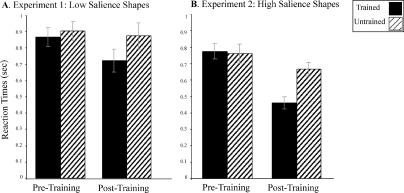
Reaction Times Reaction times before and after training for Experiments 1 (A) and 2 (B). Error bars are plus or minus the SEM.

Might the different fMRI learning effects in the LOC for low-salience (Experiment 1) and high-salience (Experiment 2) shapes be due to the subjects being less interested or paying less attention to the high- than the low-salience trained stimuli? We think that it is unlikely that the different fMRI learning effects for low- and high-salience shapes could be significantly confounded by these general attention/arousal differences across conditions for the following reasons. First, the similar behavioral learning effects for low- and high-salience shapes indicate that the observers were attentive in both tasks. Specifically, the difference in accuracy between trained and untrained shapes after training was 23.8% for low-salience shapes and 19.9% for high-salience shapes. Moreover, the observers performed the task even in the hardest condition, with untrained low-salience stimuli, as indicated by their accuracy in this condition being above chance (t_10_ = 4.23, *p* < 0.01). Further, reaction times in this condition were the slowest ( Figure 7) rather than very fast, as would be expected if the observers had given up and were simply guessing. These psychophysical data indicate that observers were engaged in the task and not responding randomly. Further, it is highly unlikely that observers could selectively choose to attend to particular conditions as trials were presented in quick succession and were randomly interleaved. Second, if the results in the LOC were simply due to task difficulty, the following pattern in the strength of fMRI responses would be expected (from high to low): untrained low saliency, untrained high saliency, trained low saliency, trained high saliency. However, the fMRI responses in the hardest (lowest accuracy) condition, untrained low-salience shapes, did not differ (F_1,7_ < 1, *p* = 0.68) from the responses in the easiest (highest accuracy) condition, trained high-salience shapes. Third, the lack of differences in the activations for trained versus untrained high-salience shapes in the early visual areas suggests that the effects observed in the LOC were not simply due to differences in general alertness or arousal across conditions that could modulate responses across all visual areas [28, 29]. Fourth, comparison of the variances after training did not show any significant differences (Levene's test, *p* > 0.05 for all ROIs) across experiments, suggesting that the different fMRI learning effects across experiments were unlikely to be due to variance differences. Finally, an additional control experiment (Figure S1) in which the observers performed a target-monitoring task [28, 29] that ensured that the observers attended similarly across conditions showed similar patterns of fMRI learning effects as those reported in Experiments 1 and 2.

Consistently, analysis of the reaction times (see Figure 7) showed that the learning effects observed in the LOC could not be due simply to differences in the duration of stimulus processing across conditions. After training, observers were slower for the untrained than the trained shapes in both experiments (Experiment 1: F_1,7_ = 136.64, *p* < 0.001); Experiment 2: (F_1,7_ = 202.35, *p* < 0.001). This effect would predict higher fMRI responses for untrained than trained stimuli in both experiments and thus could not explain the differences in the activation patterns observed across experiments. Finally, it is not likely that our learning results could be significantly confounded by eye movements. Eye movement recordings showed that the subjects were able to fixate for long periods of time, and any saccades that occurred did not differ systematically in number, amplitude, or duration for trained and untrained shapes after training (Figure S2).

## Discussion

Our experiments provide novel evidence suggesting (1) a link between behavioral improvement in shape-specific perceptual learning and neuronal plasticity in the human visual cortex, and (2) distributed plasticity mechanisms across cortical stages of visual analysis that are adaptable to natural image regularities (e.g., grouping of background elements that have the same orientation) which determine the salience of targets in cluttered scenes.

In particular, the behavioral results suggest that training enhances the observers' ability to detect shapes embedded in noisy backgrounds, providing evidence for shape-specific learning. The fMRI data suggest that these learning-dependent plasticity mechanisms, as measured by fMRI at the level of large neural populations, differ depending on the salience of the shapes. Specifically, when the shapes appeared camouflaged in cluttered backgrounds (low salience), fMRI responses were higher for trained than untrained shapes, suggesting enhanced representations of the trained shapes. However, when shapes popped out from the background (high salience), decreased fMRI responses were observed for trained shapes, suggesting sparser coding after training. Interestingly, this learning-dependent plasticity was distributed across early and higher visual areas for low-salience shapes, but was restricted to higher occipitotemporal areas for high-salience shapes. We now review these main findings in further detail.

### Behavioral Improvement and Learning-Dependent Plasticity

Several psychophysical studies have shown perceptual learning at different stages of visual analysis from features [30–36] to complex objects [3, 5, 37–39]. Further, several neurophysiological [9, 10, 12, 13, 15, 40–47] and some imaging [16–19, 48–51] studies provide evidence that behavioral improvement after training correlates with changes in neuronal sensitivity. Our findings extend our understanding of learning mechanisms, by directly testing the neural correlates of shape learning in the human visual cortex using combined psychophysical and fMRI measurements before and after training. To acquire these event-related fMRI data with high spatiotemporal resolution our investigations concentrated in occipitotemporal regions. Future studies on the cortical connectivity between these areas and prefrontal regions thought to be involved in perceptual learning [44] will provide further insights in the neural plasticity of the cortical circuits that mediate adaptive cognitive behaviors.

### Perceptual Learning Mechanisms and Shape Salience

To investigate how the visual brain learns novel objects in cluttered scenes, we chose stimuli that resemble camouflage conditions in natural images where targets are hidden due to their feature similarity with the background. Recent studies suggest that regularities (e.g., orientation similarity for neighboring elements) are characteristic of natural scenes and the primate brain has developed a network of connections that mediate integration of features based on these correlations [52–54]. In our stimuli, the orientation similarity of the target elements facilitates their grouping into global shapes. Furthermore, the uniform orientation of the background elements in the high-salience stimuli enhances the segmentation and thus the salience of the target shapes. Our findings revealed that plasticity mechanisms underlying shape learning in cluttered scenes are adaptable to these natural regularities and modulated by the perceptual saliency of the target shapes. Although our stimuli are optimal for tapping into the processing of early visual areas, these plasticity mechanisms could contribute in general to the improved detection of more natural ambiguous or low-salience targets, consistent with recent physiological investigations [45].

In particular, our findings are consistent with the idea that training with low-salience targets in cluttered scenes increases neuronal sensitivity to the target features and facilitates the detection and integration of local features into global shapes. Specifically, the learning of low-salience target shapes resulted in stronger responses to trained than untrained shapes in both early and higher visual areas. This increased neuronal sensitivity during perceptual learning [10, 11, 43, 50] has been suggested to involve increased recruitment of neurons with enhanced responses to similar features of the trained stimuli. As a result, the signal-to-noise ratio in the neural responses is increased for trained compared to untrained shapes. This process may enhance the salience of the target features, facilitating their segmentation from the background and enhancing the global integration that is important for the detection and recognition of visual targets in noise.

In contrast, when targets appear in uniform backgrounds, they are easily segmented and can be searched more efficiently [55, 56]. The lower fMRI responses observed for trained than untrained high-salience shapes are consistent with the idea that training with these pop-out targets engages smaller neural ensembles that increase their selectivity for features unique to the stimulus but most relevant for its discrimination in the context of a task. This mechanism results in sparser but more efficient representations [57] of the trained stimuli or features that are important for prompt and successful object categorization and recognition. Supporting evidence for such a mechanism comes from learning effects in the primary visual cortex after training on orientation discrimination tasks [42, 48], and the prefrontal cortex [44] where fewer neurons respond selectively to familiar than to novel objects, but they are more narrowly tuned.

Interestingly, this dissociable pattern of fMRI learning effects for low-compared to high-salience shapes provides insights into the activation patterns observed across previous learning studies. Previous studies have suggested that learning results from active long-term training [58] or rapidly from single [59] or repetitive exposure [60] to a stimulus. In our study the observers had substantial training (1,200 trials: three sessions of 400 trials each) that resulted in high-accuracy performance. It is possible that single or multiple passive exposures to target stimuli without extensive training would result in similar learning effects as those observed in our study. Taken together, previous fMRI studies show similar effects for long-term and rapid learning that depend on the nature of the stimulus representation. In particular, consistent with the fMRI activations for our low-salience shapes, enhanced responses have been observed when learning engages processes necessary for the formation of new representations, as in the case of unfamiliar [17, 61, 62], degraded [16, 63, 64] masked unrecognizable [19, 65] or noise-embedded [45, 49, 50] targets. However, when the stimulus perception is unambiguous (e.g., familiar, undegraded, recognizable targets presented in isolation), similar to our high-salience shapes, training results in more efficient processing of the stimulus features indicated by attenuated neural responses [18, 48, 62, 65–68]. Importantly, these effects are evident in areas that encode the relevant stimulus features selectively, whereas opposite activation patterns may occur in other cortical areas implicated in the task performed by the observers [44, 45, 50, 66, 68].

### Distributed Learning-Dependent Plasticity across Visual Areas

Finally, the contribution of the different visual areas in shape learning appears to depend on the salience of the target shapes. We observed fMRI learning effects in both the early visual areas and the LOC for low-salience shapes but only in the LOC for the high-salience shapes.

Previous neurophysiological [9–15, 43] and imaging [16–19] studies have implicated temporal and frontal areas in the learning of complex objects but have not investigated the contribution of early visual areas in shape learning. Early retinotopic areas have been proposed by several psychophysical studies to be involved in learning feature-discrimination tasks [30, 33, 69–73], consistent with the specificity of the learning effects for the stimulus position in the visual field [30, 36, 72–74] and the trained stimulus attribute [4, 33, 70, 71, 75]. However, neurophysiological evidence for the contribution of V1 in behavioral improvement after training on visual discrimination, remains controversial [41, 42]. There is some evidence for sharpening of orientation tuning [42] after training, but no evidence for changes in the size of the cortical representation or the receptive field properties [41, 76].

Our findings are in accordance with studies suggesting that learning is mediated by interactions between global shape-analysis mechanisms and local connections, and its neural locus could be modulated by the task context [7, 59, 70, 71, 73, 76–81]. In particular, the recognition of low-salience targets in cluttered scenes entails integration of features to global configurations and figure–ground segmentation. These processes are known to involve both early and higher visual areas [26]. The similar fMRI responses for low-salience shapes in the LOC and the early visual areas (F_5,50_ < 1, *p* = 0.77) are consistent with the involvement of both early and higher visual areas in the detection of shapes in noise. Learning has been suggested to modulate neuronal sensitivity in these areas [82] either by modulating networks of lateral interactions in the early visual areas [6, 49, 76] or via feedback connections from higher visual areas [5, 59]. However, when a salient target is present in the scene, its segmentation is easily achieved and learning may contribute to the representation of the critical features for shape recognition. Thus, learning tunes the representations of global shapes that are known to involve higher occipitotemporal areas [27]. Our results showing stronger fMRI responses for high-salience shapes in the LOC than in the early visual areas (F_5,35_ = 1.91, *p* < 0.05) are consistent with processing of salient global shapes in the LOC.

Consistent with this evidence for distributed cortical plasticity, recent psychophysical studies [59, 83, 84] have proposed a reverse hierarchy theory (RHT) of perceptual learning. This theory proposes that learning begins at high-level areas for easy tasks and proceeds to early retinotopic areas that have higher resolution necessary for finer and more difficult discriminations. Although fMRI studies lack the temporal resolution necessary for testing this proposal, our findings are consistent with plasticity mechanisms in early visual areas that mediate learning in difficult and fine tasks (i.e., detection of low-salience rather than high-salience shapes) [55, 56, 59]. It is possible that these learning effects in early visual areas are the result of feedback from higher areas. As the discrimination of low-salience shapes improves with training [6, 78, 79], higher shape-related areas increase their responses and enhance the processing of the trained shape features in the early visual areas that have fine spatial resolution necessary for the detection of targets in noise. Finally, this theory makes interesting predictions for learning specificity to the trained features in easy tasks, in contrast to generalization across image changes in difficult tasks [85]. Testing these predictions for specificity, feedback triggered by single vs. repeated exposure, and long-lasting plasticity would be of interest in future studies.

### Conclusions

In summary, our findings propose that the human brain learns novel objects in complex scenes by reorganizing shape processing across early and higher cortical stages of visual analysis. Interestingly, this learning-dependent plasticity is implemented by mechanisms that are adaptable to the target scene. That is, the visual brain appears to take advantage of natural image correlations that determine the target distinctiveness in a scene while learning novel object targets. Our study provides novel neuroimaging evidence that this opportunistic learning [5] of salient targets in natural scenes is mediated by sparser feature coding at higher stages of visual analysis, whereas learning of camouflaged targets is implemented by bootstrapped mechanisms [5] that enhance the segmentation and recognition of ambiguous targets in both early and higher visual areas.

## Materials and Methods

### Subjects

Twenty-six students from the University of Tübingen participated in the experiment. Seven observers (two for Experiment 1 and five for Experiment 2) were excluded due to either excessive head movement or poor performance in the psychophysical task (accuracy was two standard deviations below the mean). All subjects provided informed consent.

### Stimuli and procedure

Thirty-two symmetrical and 32 asymmetrical shapes were rendered with collinear Gabor elements (0.55°) and embedded in backgrounds of randomly-positioned Gabors (0.55°), as described previously [26]. Each stimulus covered an area 14.35° × 14.35° (average shape area: 7.72° × 7.78°) and was presented 0.19° to the left or right of fixation. Two types of background were used: (1) randomly positioned and oriented Gabors (low salience) or (2) randomly positioned but uniformly oriented Gabors (high salience). A pilot psychophysical experiment showed that detection of symmetrical and asymmetrical shapes in these stimuli was of similar difficulty. To ensure that subjects learned the shapes and not simply the background configuration, the arrangement of the background elements differed on every trial and the position of the shape target elements was jittered. Each observer was trained on a unique set of four symmetrical and four asymmetrical shapes that were presented on all days of the experiment. Observers performed a two-alternative forced choice (2AFC) task. On every trial, one symmetrical and one asymmetrical stimulus were presented on either side of the fixation point. Observers indicated (by pressing a button) which side of the display contained the symmetrical shape while maintaining central fixation. This 2AFC task was chosen for two reasons: (1) to encourage the observers to compare the two stimuli presented in a trial and improve their performance by learning to discriminate between shapes, and (2) to avoid biases that are observed in detection tasks when a single stimulus is presented in a trial. One possible limitation of this task is that the observers could adopt a strategy in which they only paid attention to one side of the screen. Such a strategy would make it easy for the observers to perform the 2AFC task even before training. However, the observers' poor performance in the 2AFC task before training suggests that it is unlikely that observers relied on such a strategy.Furthermore, limiting attention to one side of the fixation point could result in hemispheric asymmetries in the fMRI data. However, we did not observe any differences in the pattern of fMRI data between hemispheres.

### Pre-training scanning session (Day 1)

On the first day of the experiment, observers performed the 2AFC task without feedback in the scanner. This scanning session consisted for four different event-related runs in which the observers were presented with low-salience (Experiment 1), or high-salience (Experiment 2) trained stimuli (i.e., that were to become trained) and untrained stimuli (i.e., that were to remain untrained). In particular, on each run observers viewed a set of trained (four symmetrical and four asymmetrical) stimuli and a set of untrained (four symmetrical and four asymmetrical) stimuli. Each run consisted of one epoch of experimental trials and two 8-s fixation epochs (one at start, one at end). Each run had 25 experimental trials for each condition (trained and untrained) and 25 fixation trials; that is, a total of 75 trials. A new trial began every 3 s and consisted of a stimulus image presented for 300 ms and a blank interval of 2,700 ms. As in previous studies [26], the order of presentation was counterbalanced so that trials from each condition, including the fixation condition, were preceded equally often by trials from each of the other conditions. In total, 100 trials were collected for each experimental condition across the four runs in each scanning session.

### Psychophysical training sessions (Days 2–4)

**Figure 8:**
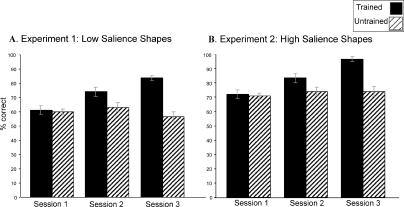
Behavioral Data during Training Sessions Psychophysical data (percent correct) during the three training sessions. Data are shown for trained and untrained shapes in which the observers were tested without feedback at the end of each training session. Statistical analysis of the data showed that the observers' performance improved for trained, but not untrained, shapes across training sessions. (A) In Experiment 1, no significant differences between trained and untrained stimuli were observed for session 1 (F_1,20_ = 1.66 , *p* = 0.21) but increasing differences were observed for sessions 2 (F_1,20_ = 19.57, *p* < 0.001) and 3 (F_1,20_ = 47.79, *p* < 0.001). (B) Similarly, in Experiment 2, a significant effect (F_1,14_ = 27.30, *p* < 0.01) of familiarity (trained vs. untrained shapes) was observed across training sessions.

Observers were trained with four symmetrical and four asymmetrical shapes from their unique trained-stimulus set (one set per observer) in either the low-salience (Experiment 1) or the high-salience (Experiment 2) target condition for three consecutive days. Each session consisted of 400 training trials (total trials across sessions: 1,200) during which error feedback was given. At the end of each training session, observers performed a short test of 96 trials (no feedback) in which trained shapes were intermixed with untrained shapes. As shown in Figure 8, the observers' performance improved across training sessions for the trained, but not the untrained, stimuli, suggesting shape-specific learning rather than general improvement in the 2AFC task.

### Post-training scanning session (Day 5)

Observers were tested in the scanner on the same set of stimuli with which they were presented in the pre-training scanning session. The same procedure was followed as in the pre-training scanning session.

### Imaging

Observers were scanned in a 3T Siemens scanner at the University Clinic in Tübingen. Gradient echo pulse sequences were used (TR = 1 s, TE = 40 ms for event related runs; TR = 2 s, TE = 90 ms for localizer runs. Data were collected with a head coil from eleven axial slices (3 × 3 mm in-plane resolution, 5-mm thickness) that covered the occipitotemporal cortical regions.

### Data analysis

Psychophysical data were analyzed with repeated measures ANOVA on test session (before training, after training), and familiarity (trained, untrained) for each experiment. Contrast analysis followed significant interactions between these factors.

The fMRI data were analyzed with Brain Voyager, as described previously [26]. For each individual subject we identified the early visual areas and the LOC as cortical ROIs (see Figure 2). The LOC was defined as the set of all voxels in the ventral occipitotemporal cortex that were activated more strongly (*p* < 10^−4^) by intact than scrambled images of objects presented in two blocked-design runs [26]. Two subregions of the LOC were identified [19]: the LO at the posterior part of the inferior-temporal sulcus and the pFs in the posterior fusiform gyrus. Early ventral visual areas were identified using standard retinotopic mapping techniques [26].

For each individual subject we extracted time-course data from each ROI and for each condition (Figures S3 and S4). Fitting the time course data with the hemodynamic response function and ANOVA analysis across time points indicated that peak time fMRI responses occurred at 4 and 5 s after trial onset (Figures S5 and S6). For statistical analysis of differences between conditions in the average fMRI responses at these time points we used repeated-measures ANOVA on test session (before training and after training), familiarity (trained and untrained) and ROI (V1, V2, Vp, V4, LO, and pFs). Contrast analysis followed significant interactions between these factors.

## Supporting Information

Figure S1 Attentional Control Experiment: Post-Training Test fMRI responses after training obtained when observers performed a target-monitoring task while being presented with either low-salience (A) (three observers) or high-salience (B) (three observers) shapes, as in Experiments 1 and 2. The observers were instructed to press a button when a prespecified target shape appeared in a trial. This task ensured that the observers paid attention across all conditions similarly, as the target's appearance was rare (∼15% of trials) but of similar frequency across conditions. Detection of this target was of comparable difficulty for low-salience (79%) and high-salience (81%) shapes. Similar patterns of fMRI data were observed in this control experiment as in Experiments 1 and 2.
(257 KB PDF).

Figure S2 Eye Movement Controls Eye movements of six subjects in Experiment 1 and Experiment 2 were recorded (Eye-Link video based system, 250-Hz sample rate) for the pre-training and post-training sessions. We compared eye position and saccades between trained and untrained shapes in each experiment. In the pre-training session, the average number of saccades (Experiment 1: horizontal F_2,4_ < 1; *p* = 0.77, vertical F_2,4_ < 1; *p* = 0.45; Experiment 2: horizontal F_2,4_ < 1; *p* = 0.43, vertical F_2,4_ < 1; *p* = 0.36) and amplitude (Experiment 1: horizontal F_2,4_ < 1;p = 0.88, vertical F_2,4_ < 1; *p* = 0.37; Experiment 2: horizontal F_2,4_ < 1; *p* = 0.58, vertical F_2,4_ < 1; *p* = 0.67) did not differ between experimental conditions and the fixation condition. Data are shown for the post-training session, in which psychophysical and fMRI differences between trained and untrained stimuli were observed. Panels A–B show that the histograms of the horizontal eye position for each condition and experiment were peaked and centered on the fixation at zero degrees. Similar histograms of the vertical eye position were centered on the fixation but less sharply peaked. (This was probably due to observed drift in the vertical position signal over the course of the recordings). No significant differences were observed in the mean eye position between fixation, trained and untrained conditions for low-salience shapes (x position F_2,4_ = 1.29; *p* = 0.36, y position F_2,4_ = 3.05; *p* = 0.15) and high-salience shapes (x position F_2,4_ = 1.23; *p* = 0.38, y position F_2,4_ < 1; *p* = 0.95). Furthermore, the average number of saccades (panel C) was similar in both experiments and did not differ significantly for trained and untrained shapes (low salience F_1,2_ < 1; *p* = 0.94; high salience F_1,2_ < 1; *p* = 0.70). The amplitude (low salience F_1,2_ < 1; *p* = 0.47; high salience F_1,2_ < 1; *p* = 0.99) and duration (low salience F_1,2_ < 1; *p* = 0.32; high salience F_1,2_ < 1; *p* = 0.63) of these saccades did not differ significantly for trained and untrained shapes (panels D–G).
(428 KB PDF).

Figure S3 Time Course of the fMRI Responses I: Experiment 1 These figures illustrate the time course (0–10 s after trial onset) of the fMRI responses for all ROIs in Experiment 1 (Figure S3) and Experiment 2 (Figure S4) before (A) and after (B) training. Error bars are plus or minus the standard error of the mean (SEM). As previously described [26], for each event-related scan, the fMRI responses were extracted by averaging the data from all voxels within each subject's ROIs. We averaged the signal intensity across trials in each condition at each time point and converted these to percent signal change relative to fixation. We then averaged each condition's time course across scans for each subject and then across subjects.
Because of the hemodynamic lag in the fMRI response, the peak in overall response and, therefore, the differences across conditions are expected to occur at a lag of several seconds after stimulus onset [86– 88]. In accordance with the hemodynamic response properties, an ANOVA between familiarity (trained, untrained) and time point (0–10 s after trial onset) for each ROI showed statistical differences for time point 4 (e.g., LOC: Experiment 1 (F_1,100_ = 8.28, *p* < 0.01), Exp 2 (F_1,70_ = 5.31, *p* < 0.05)), and time point 5 (e.g., LOC: Experiment 1 (F_1,100_ = 19.75, *p* < 0.001), Experiment 2 (F_1,70_ = 6.01, *p* < 0.05)), but not at trial onset, i.e., time point zero (e.g., LOC: Experiment 1 (F_1,100_ < 1, *p* = 0.59), Experiment 2 (F_1,70_ = 2.64, *p* = 0.14)). Results were similar in the other ROIs in that no significant differences were observed at trial onset in early visual areas for low-salience (Experiment 1: F_1,100_ = 2.23, *p* = 0.15) or high-salience (Experiment 2: F_1,70_ = 1.63, *p* = 0.23) shapes.
(711 KB PDF).

Figure S4 Time Course of the fMRI Responses II: Experiment 2 These figures illustrate the time course (0–10 s after trial onset) of the fMRI responses for all ROIs in Experiment 1 (Figure S3) and Experiment 2 (Figure S4) before (A) and after (B) training. Error bars are plus or minus the standard error of the mean (SEM). As previously described [26], for each event-related scan, the fMRI responses were extracted by averaging the data from all voxels within each subject's ROIs. We averaged the signal intensity across trials in each condition at each time point and converted these to percent signal change relative to fixation. We then averaged each condition's time course across scans for each subject and then across subjects.
Because of the hemodynamic lag in the fMRI response, the peak in overall response and, therefore, the differences across conditions are expected to occur at a lag of several seconds after stimulus onset [86– 88]. In accordance with the hemodynamic response properties, an ANOVA between familiarity (trained, untrained) and time point (0–10 s after trial onset) for each ROI showed statistical differences for time point 4 (e.g., LOC: Experiment 1 (F_1,100_ = 8.28, *p* < 0.01), Exp 2 (F_1,70_ = 5.31, *p* < 0.05)), and time point 5 (e.g., LOC: Experiment 1 (F_1,100_ = 19.75, *p* < 0.001), Experiment 2 (F_1,70_ = 6.01, *p* < 0.05)), but not at trial onset, i.e., time point zero (e.g., LOC: Experiment 1 (F_1,100_ < 1, *p* = 0.59), Experiment 2 (F_1,70_ = 2.64, *p* = 0.14)). Results were similar in the other ROIs in that no significant differences were observed at trial onset in early visual areas for low-salience (Experiment 1: F_1,100_ = 2.23, *p* = 0.15) or high-salience (Experiment 2: F_1,70_ = 1.63, *p* = 0.23) shapes.
(711 KB PDF).

Figure S5Fits to the Time Course Data I: Experiment 1To confirm the peak points obtained from ANOVA analysis, we fit the data using two Gaussians (one for the initial response and one for the undershoot) and a baseline:

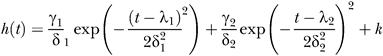

from Kruggel and von Cramon [89] where the hemodynamic response function (h) over time (t) is modeled as the sum of two Gaussians, each of which depends on gain (γ), dispersion (δ), a temporal lag (λ), and a baseline parameter (k). Fits for the fMRI responses across all areas for trained and untrained stimuli are shown for Experiment 1 (Figure S5) and Experiment 2 (Figure S6) before (A) and after (B) training. These fits showed the following peak time points for each condition: Experiment 1 (before training: trained: 4.25, untrained: 4.23; after training: trained: 4.27, untrained: 4.24); Experiment 2 (before training: trained: 4.24, untrained: 4.29; after training: trained: 4.23, untrained: 4.21). This analysis confirmed the selection of time points 4 and 5 as the peak points of the fMRI time courses. Therefore the average response at these peak points was taken as the measure of response magnitude for each condition in subsequent analyses. Analysis of time points 2–6 s after stimulus onset or the area under the curve showed the same pattern of results as reported in the paper.(363 KB PDF).

Figure S6Fits to the Time Course Data II: Experiment 2To confirm the peak points obtained from ANOVA analysis, we fit the data using two Gaussians (one for the initial response and one for the undershoot) and a baseline:

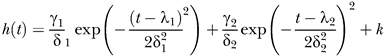

from Kruggel and von Cramon [89] where the hemodynamic response function (h) over time (t) is modeled as the sum of two Gaussians, each of which depends on gain (γ), dispersion (δ), a temporal lag (λ), and a baseline parameter (k). Fits for the fMRI responses across all areas for trained and untrained stimuli are shown for Experiment 1 (Figure S5) and Experiment 2 (Figure S6) before (A) and after (B) training. These fits showed the following peak time points for each condition: Experiment 1 (before training: trained: 4.25, untrained: 4.23; After training: trained: 4.27, untrained: 4.24); Experiment 2 (Before training: trained: 4.24, untrained: 4.29; after training: trained: 4.23, untrained: 4.21). This analysis confirmed the selection of time points 4 and 5 as the peak points of the fMRI time courses. Therefore the average response at these peak points was taken as the measure of response magnitude for each condition in subsequent analyses. Analysis of time points 2–6 s after stimulus onset or the area under the curve showed the same pattern of results as reported in the paper.(366 KB PDF).

## Abbreviations

2AFC: two-alternative forced choice
fMRI: functional magnetic resonance imaging
LO: lateral occipital
LOC: lateral occipital complex
pFs: posterior fusiform sulcus
ROI: region of interest
SEM: standard error of the mean
