# Supplementary material for: Distributed Neural Plasticity for Shape Learning in the Human Visual Cortex
Source: PLoS Biol. 2005 Jun 7;3(7):e204. doi: 10.1371/journal.pbio.0030204 (PMC1150289; doi:10.1371/journal.pbio.0030204)

A. Experiment 2:fMRI responses:Pre-TrainingTest

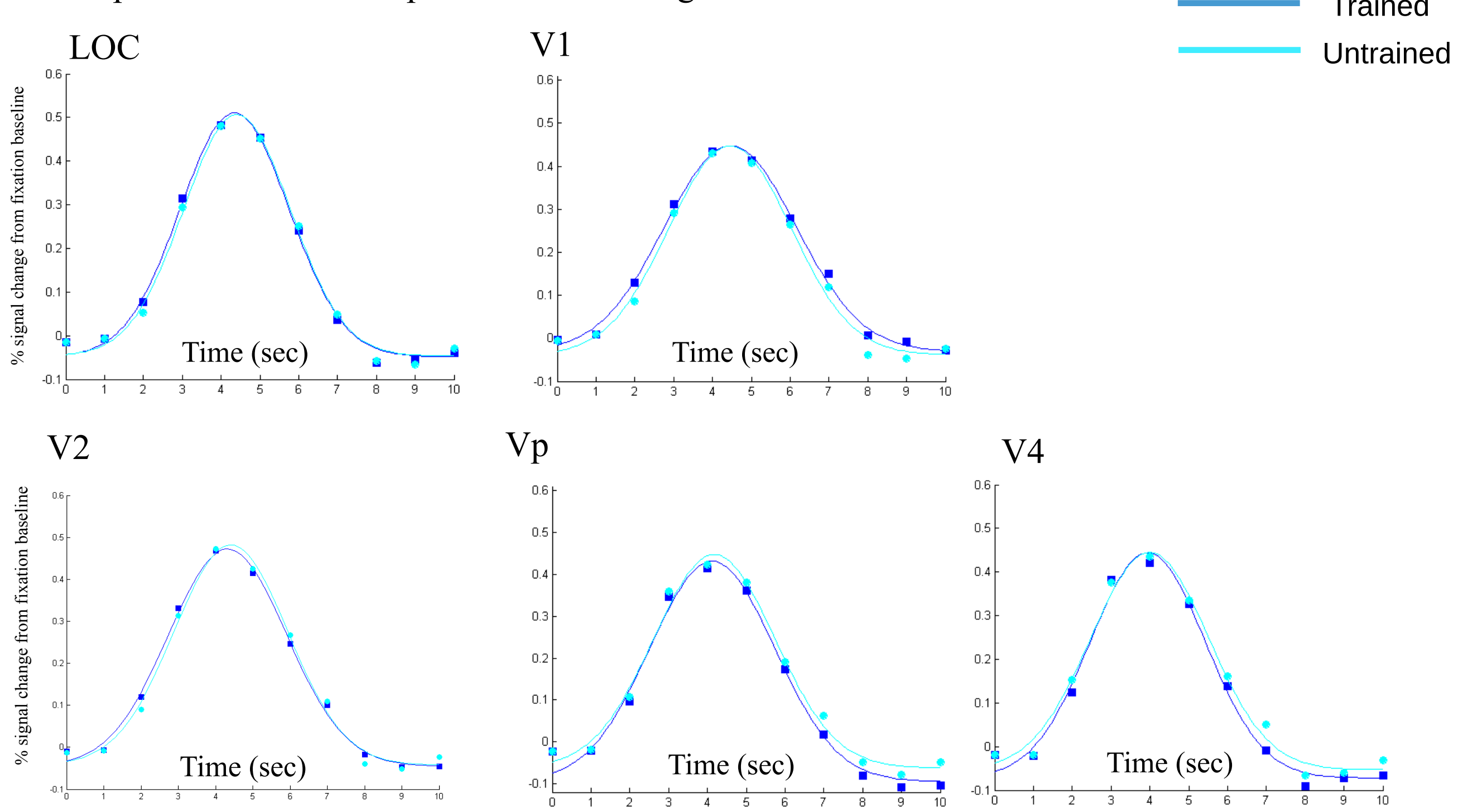

B. Experiment 2:fMRI responses:Post-TrainingTest

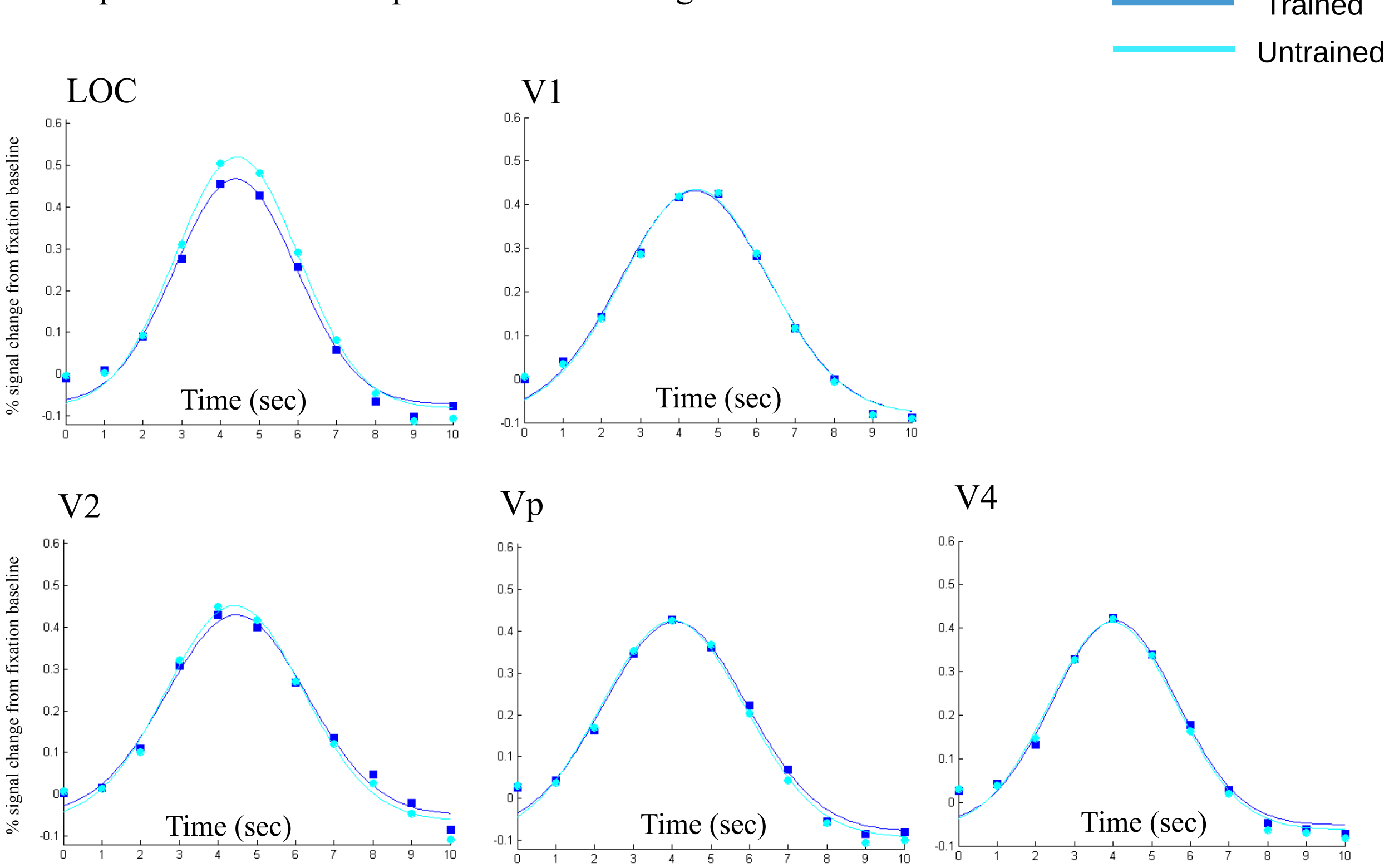

Supplement: Supplementary file 6 [file pbio.0030204.sg006.pdf]
